# Spectroscopy of short-lived radioactive molecules

**DOI:** 10.1038/s41586-020-2299-4

**Published:** 2020-05-27

**Authors:** R. F. Garcia Ruiz, R. Berger, J. Billowes, C. L. Binnersley, M. L. Bissell, A. A. Breier, A. J. Brinson, K. Chrysalidis, T. E. Cocolios, B. S. Cooper, K. T. Flanagan, T. F. Giesen, R. P. de Groote, S. Franchoo, F. P. Gustafsson, T. A. Isaev, Á. Koszorús, G. Neyens, H. A. Perrett, C. M. Ricketts, S. Rothe, L. Schweikhard, A. R. Vernon, K. D. A. Wendt, F. Wienholtz, S. G. Wilkins, X. F. Yang

**Affiliations:** 1 CERN, Geneva, Switzerland; 2 Massachusetts Institute of Technology, Cambridge, MA USA; 3 Fachbereich Chemie, Philipps-Universität Marburg, Marburg, Germany; 4 Department of Physics and Astronomy, The University of Manchester, Manchester, UK; 5 Laboratory for Astrophysics, Institute of Physics, University of Kassel, Kassel, Germany; 6 KU Leuven, Instituut voor Kern- en Stralingsfysica, Leuven, Belgium; 7 Photon Science Institute, The University of Manchester, Manchester, UK; 8 Department of Physics, University of Jyväskylä, Jyväskylä, Finland; 9 Institut de Physique Nucleaire d’Orsay, Orsay, France; 10 NRC ‘Kurchatov Institute’-PNPI, Gatchina, Russia; 11 Institut für Physik, Universität Greifswald, Greifswald, Germany; 12 Institut für Physik, Johannes Gutenberg-Universität Mainz, Mainz, Germany; 13 School of Physics and State Key Laboratory of Nuclear Physics and Technology, Peking University, Beijing, China

**Keywords:** Electronic structure of atoms and molecules, Exotic atoms and molecules, Experimental nuclear physics

## Abstract

Molecular spectroscopy offers opportunities for the exploration of the fundamental laws of nature and the search for new particle physics beyond the standard model^1–4^. Radioactive molecules—in which one or more of the atoms possesses a radioactive nucleus—can contain heavy and deformed nuclei, offering high sensitivity for investigating parity- and time-reversal-violation effects^5,6^. Radium monofluoride, RaF, is of particular interest because it is predicted to have an electronic structure appropriate for laser cooling^6^, thus paving the way for its use in high-precision spectroscopic studies. Furthermore, the effects of symmetry-violating nuclear moments are strongly enhanced^5,7–9^ in molecules containing octupole-deformed radium isotopes^10,11^. However, the study of RaF has been impeded by the lack of stable isotopes of radium. Here we present an experimental approach to studying short-lived radioactive molecules, which allows us to measure molecules with lifetimes of just tens of milliseconds. Energetically low-lying electronic states were measured for different isotopically pure RaF molecules using collinear resonance ionisation at the ISOLDE ion-beam facility at CERN. Our results provide evidence of the existence of a suitable laser-cooling scheme for these molecules and represent a key step towards high-precision studies in these systems. Our findings will enable further studies of short-lived radioactive molecules for fundamental physics research.

## Abstract (web summary)

Measurements of low-energy electronic states of radium monofluoride validate predictions of the use of this short-lived radioactive molecule in exploring fundamental physics and provide evidence of its suitability for laser cooling.

## Main

Molecular systems provide a versatile physical environment in which to study the fundamental symmetries of nature and the interactions and properties of subatomic particles^1,2,12,13^. Among the four known fundamental forces, the weak force is the only one that is known to violate symmetry with respect to spatial inversion of all particle coordinates (known as parity violation), giving rise to various intriguing phenomena. Some of these parity-violating effects have been measured with high accuracy in atomic systems^13–15^, contributing to the most stringent low-energy tests of the Standard Model of particle physics. In certain molecules, effects resulting from both parity violation (P-odd) and time-reversal violation (T-odd) are considerably enhanced with respect to atomic systems^5,7,8,13,16^, offering the means to explore unknown aspects of the fundamental laws of physics. The strengths of these interactions scale with atomic number, nuclear spin and nuclear deformation, and so molecular compounds of heavy radioactive nuclei are predicted to exhibit unprecedented sensitivity, with an enhancement of more than two orders of magnitude for effects that are P-odd or simultaneously P- and T-odd^5–8,17–20^.

However, the experimental knowledge of radioactive molecules is scarce^21^, and quantum chemistry calculations often constitute the only source of information. Molecules possess complex quantum level structures, which renders spectroscopy of their structure considerably more challenging compared to atoms. Moreover, major additional experimental challenges must be overcome to study molecules containing heavy and deformed nuclei, which can have lifetimes of just a few milliseconds. These radioactive nuclei are very rare in nature or do not occur naturally and so must be produced artificially at specialized facilities, such as at the Isotope Separator On-line Device (ISOLDE) at CERN. Furthermore, molecules containing short-lived isotopes can only be produced in quantities smaller than 10^−8^ g (typically with rates of less than 10^6^ particles s^−1^). Thus, spectroscopic studies require particularly sensitive experimental techniques adapted to the properties of radioactive ion beams and the conditions present at radioactive-beam facilities. Here, we present an approach for performing laser spectroscopy of short-lived radioactive molecules, using the highly sensitive collinear resonance ionization method^22^. These results provide the first spectroscopic information of RaF, including isotopologues composed of radioactive isotopes with lifetimes as short as a few days. To our knowledge, this is the first laser spectroscopy study performed on a molecule containing a short-lived isotope. Moreover, this experimental scheme can be applied to study other radioactive molecules, even those composed of isotopes with lifetimes as short as a few tens of milliseconds.

Since the direct cooling of diatomic molecules with lasers^23^ was experimentally demonstrated^24^, there has been a wealth of studies on laser-cooling techniques and applications in molecular physics^25–31^. In contrast to other heavy-atom molecules, RaF is predicted to have highly closed excitation and re-emission optical cycles, which would make it ideal for laser cooling and trapping^6^. Moreover, owing to the recently discovered pear-shaped nuclear deformation of certain radium isotopes^11^, the interactions of the electrons with the P-odd nuclear anapole moment as well as with the P,T-odd nuclear Schiff and magnetic quadrupole moments are predicted to be enhanced by more than two orders of magnitude^4,5,19,32^. Hence, these molecules could provide a unique environment in which to measure these symmetry-violating nuclear moments.

## Experimental scheme

**Fig. 1:**
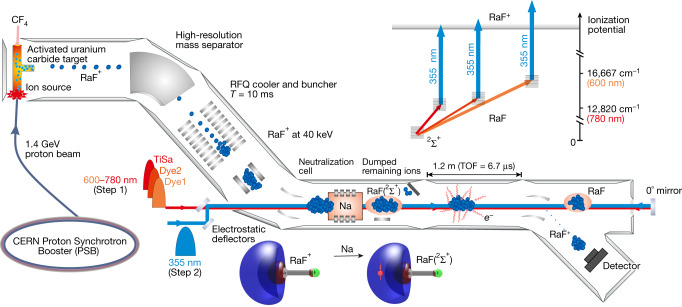
Experimental scheme for the production and study of short-lived radioactive molecules. Radioactive radium isotopes were created by impinging 1.4-GeV protons from the CERN Proton Synchrotron Booster (PSB) on a uranium carbide (UC_*x*_) target. Radium monofluoride cations (RaF^+^) were produced by passing tetrafluoromethane (CF_4_) gas through the activated UC_*x*_ target at 1,300 °C. Molecular ions were extracted from the source, mass-selected and injected into a helium-filled RFQ trap, where they were accumulated for 10 ms. Bunches of molecular ions were extracted and neutralized in flight by charge exchange with neutral sodium atoms. Neutral RaF molecules were overlapped with different laser beams (step 1, TiSa, Dye1 and Dye2, and step 2, a 355-nm laser; see Methods section ‘Laser setup’) in a collinear geometry. Resonantly reionized molecules were deflected onto a particle detector. The resonance ionization scheme is shown at top right. At bottom, molecular orbitals are shown schematically. Nuclear positions within the molecules are coarsely indicated by a grey sphere (Ra) and green sphere (F), and the sigma bond between the atoms is indicated by the grey cylinders. Further details are provided in ‘Experimental scheme’.

Figure 1 shows a diagram of the experimental setup used to produce and study the RaF molecules. As a first step, radium isotopes were produced by diffusion out of an irradiated target (see Methods section ‘Production of RaF molecules’). RaF^+^ molecular ions were formed upon injection of CF_4_ gas into the target environment. The molecular ions were extracted from the ion source by applying an electrostatic field, and molecules containing one specific radium isotope were selected with a high-resolution magnetic mass separator (Δ*m*/*m* ≈ 1/2,000). The ions were collisionally cooled in a radio-frequency quadrupole (RFQ) trap filled with helium gas at room temperature (about 300 K). After up to 10 ms of cooling time, bunches of RaF^+^ with a 4-μs temporal width were released and accelerated to 39,998(1) eV, before entering into the Collinear Resonance Ionisation Spectroscopy (CRIS) setup^22,33,34^. At the CRIS beam line, the ions were first neutralized in-flight by passing through a collision cell filled with a sodium vapour, inducing charge exchange according to the reaction RaF^+^ + Na → RaF + Na^+^. As the ionization energy of RaF is estimated to be close to that of sodium (5.14 eV)^35^, the neutralization reaction dominantly populates the RaF X^2^Σ^+^ electronic ground state. Molecular pseudo-orbitals obtained from one-component open-shell (neutral) or closed-shell (ion) restricted Hartree–Fock calculations with an energy-consistent effective core potential on radium are shown schematically in Fig. 1 (bottom). The lowest unoccupied molecular orbital in RaF^+^, which is mainly of non-bonding character, becomes occupied by an unpaired electron (symbolized in Fig. 1 by a red sphere together with an arrow representing the electron spin) upon neutralization. This is shown schematically as an isodensity, with lobes in slightly transparent blue and transparent red indicating different relative phases of the single-electron wavefunction.

After the charge-exchange reaction, non-neutralized RaF^+^ ions were deflected out of the beam, and the remaining bunch of neutral RaF molecules was overlapped in time and space by several (pulsed) laser beams in a collinear arrangement, along the ultrahigh-vacuum (10^−10^ mbar) interaction region of 1.2-m length. Laser pulses (step 1) of tunable wavelength were used to resonantly excite the transition of interest, and a high-power 355-nm laser pulse (step 2) was used to subsequently ionize the excited RaF molecules into RaF^+^ (see Fig. 1, top). The resonantly ionized molecules were then separated from the non-ionized molecules by deflecting the ions onto a particle detector. When the excitation laser is on resonance with a transition in the molecule (step 1 in Fig. 1), the second laser pulse ionizes the molecule, producing a signal at the detector. Molecular excitation spectra were obtained by monitoring the ion counts as a function of the wavenumber of the first laser.

Only theoretical predictions were available for the excitation energies of RaF, and so finding the transition experimentally required scanning a large wavelength range (>1,000 cm^−1^). The prediction for the A^2^Π_1/2_−X^2^Σ^+^ (0, 0) transition, for example, was 13,300 cm^−1^, with an accuracy estimated to be within 1,200 cm^−1^ (refs. ^6,32^). Given the bandwidths of the commonly available lasers (<0.3 cm^−1^), the scan of such a large wavelength region on samples produced at rates below 10^6^ molecules s^−1^ represented a major experimental challenge. To optimize the search of molecular transitions, three broadband lasers were scanned simultaneously and both collinearly and anti-collinearly (see Methods section ‘Laser setup’).

## Results

**Fig. 2:**
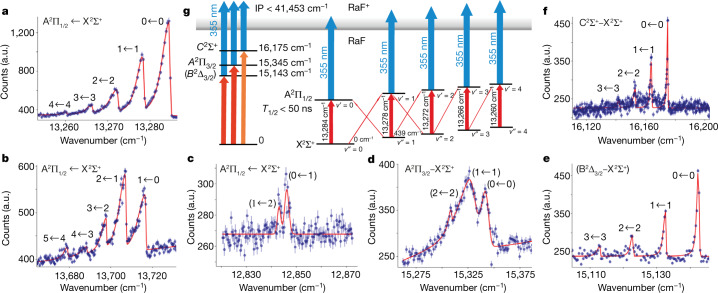
Examples of vibronic spectra measured for ^226^RaF. **a**–**f**, The counts on the particle detector were measured as a function of the laser wavenumber of the resonant step. A fixed wavelength (355 nm) was used for the ionization step. **a**, The observed peaks corresponding to the vibronic spectra of the Δ*v* = 0 band system of *v*″ = 0, 1, 2, 3, 4, scanned by the grating Ti:sapphire laser. **b**, **c**, The pulsed dye laser was used to scan electronic transitions in different wavelength ranges: the Δ*v* = +1 band system of the A^2^Π_1/2_ ← X^2^Σ^+^ transition with *v*″ = 0, 1, 2, 3, 4 (**b**) and the (*v*′, *v*″) = (0, 1) and (1, 2) band. **d**–**f**, The corresponding transitions to other electronic states: A^2^Π_3/2_ ← X^2^Σ^+^ (**d**), B^2^Δ_3/2_ ← X^2^Σ^+^ (tentatively assigned; **e**) and C^2^Σ^+^ ← X^2^Σ^+^ (**f**). The shape of the spectra is due to population distribution of different rotational states. The solid lines show the fit with skewed Voigt profiles. **g**, Scheme of the molecular energy levels. The estimated upper limit of the ionization potential (IP) is indicated. Three essential properties for laser cooling of RaF molecules were identified: 1) the short lifetime of the excited states ^2^Π_1/2_ (*T*_1/2_ < 50 ns), which will allow for the application of strong optical forces; 2) dominant diagonal transitions, (Δ*v* = 0)/(Δ*v* = ±1, Δ*v* = 0) > 0.97, indicating a large diagonal Franck–Condon factor; and 3) the expected low-lying electronic states B^2^Δ_3/2_, A^2^Π_3/2_ and C^2^Σ^+^ were found to be above the A^2^Π_1/2_ states, which will enable efficient optical-cooling cycles. Wavenumbers in the spectra are given in the rest frame of the molecule. In **a**–**f**, the error bars show the statistical uncertainties (1 standard deviation) for the number of resonantly ionized molecules obtained within each laser frequency interval.

The predicted region for the A^2^Π_1/2_ ← X^2^Σ^+^ transition was scanned at a speed of 0.06 cm^−1^ s^−1^, covering a range of 1,000 cm^−1^ in about 5 h, using the six simultaneously applied scanning regions. After a few hours of scanning on a beam of ^226^RaF, a clear sequence of vibronic absorption signals was recorded. The measured spectrum assigned to the (*v*′, *v*″) vibrational transitions (0, 0), (1, 1), (2, 2), (3, 3) and (4, 4) of the A^2^Π_1/2_−X^2^Σ^+^ band system is shown in Fig. 2a. Weaker band structures, that were found at about +440 cm^−1^ and −440 cm^−1^ with respect to the (0, 0) band, were assigned to the Δ*v* = ±1 transitions (*v*′, *v*″) = (1, 0), (2, 1), (3, 2), (4, 3), (5, 4) and (*v*′, *v*″) = (0, 1), (1, 2), respectively (Fig. 2b, c). The quantum number assignment for Δ*v* = −1 is tentative, owing to the highly dense structure of overlapping vibronic bands.

In addition to the A^2^Π_1/2_−X^2^Σ^+^ band system, we found spectroscopic signatures of electronic transitions to higher-lying states. Some examples of recorded spectra are shown in Fig. 2d–f, along with the energy-level scheme. We assign the observed transitions as follows: 1) The band system around 15,325 cm^−1^ (Fig. 2d) is attributed to the A^2^Π_3/2_−X^2^Σ^+^ transition, owing to the complex rovibrational structure expected to arise from the intense satellites that are possible in these transitions. Because the bands are comparatively strong, they are assigned to the Δ*v* = 0 band system. Although the individual assignments to vibrational transitions must be considered to be tentative, as per the congested structure of the Franck–Condon profile, the Δ*v* = 0 assignment is substantiated because no additional structure was located within a relative range of −400 to +400 cm^−1^. The band system located around 15,143 cm^−1^ (Fig. 2e) is tentatively assigned to the B^2^Δ_3/2_−X^2^Σ^+^ transition by virtue of the good agreement with the computed excitation energies to the Ω = 3/2 state of mixed Δ/Π character^6,32^. This mixing provides intensity to the one-photon transition from a Σ state into the Δ manifold. The computed Born–Oppenheimer potentials for this Ω = 3/2 state and the electronic ground state are, however, highly parallel, which would suggest a sparser Franck–Condon profile than was observed experimentally. However, we note that the related B^2^Δ_3/2_−X^2^Σ^+^ transition in BaH and BaD was reported to have a perturbed character owing to mixing between electronic levels^36^. Thus, in the present case, a vibrational profile that is richer than expected from adiabatic potentials cannot be ruled out a priori. The band system with origin at 16,175 cm^−1^ (Fig. 2f) is assigned to the C^2^Σ^+^−X^2^Σ^+^ transition on the basis of the observed Franck–Condon profile, which is in good agreement with the computed harmonic vibrational energy spacings as well as the expected intensity distribution, and is in a wavenumber region that is only slightly lower than predicted^6,32^. All measured and assigned vibronic bands of the four electronic transitions are listed in Table 1.

**Table 1:** Measured vibronic transitions of ^226^RaF from the X^2^Σ^+^ electronic ground state to the excited A^2^Π and B^2^Δ states

Transition	*v*′ ← *v*″	${\boldsymbol{\Delta }}\tilde{{\boldsymbol{\nu }}}$ (cm^−1^)
A^2^Π_1/2_ ← X^2^Σ^+^	0−0	13,284.7(5)
	1−1	13,278.5(5)
	2−2	13,272.4(5)
	3−3	13,266.4(10)
	4−4	13,260.2(10)
	1−0	13,716.9(5)
	2−1	13,707.4(5)
	3−2	13,698.0(5)
	4−3	13,688.6(10)
	5−4	13,679.4(10)
	(0−1)	12,846.3(10)
	(1−2)	12,843.1(10)
(B^2^Δ_3/2_ ← X^2^Σ^+^)	0−0	15,142.7(5)
	1−1	15,132.8(10)
	2−2	15,123.0(10)
	3−3	15,113.2(10)
A^2^Π_3/2_ ← X^2^Σ^+^	(0−0)	15,344.6(50)
	(1−1)	15,325.0(80)
	(2−2)	15,309.4(100)
C^2^Σ^+^ ← X^2^Σ^+^	0−0	16,175.2(5)
	1−1	16,164.2(5)
	2−2	16,153.4(5)
	3−3	16,142.4(10)

The values indicate the band head positions.

Combined statistical and systematic uncertainties are included in parentheses.

The B^2^Δ_3/2_ ← X^2^Σ^+^ assignment is tentative.

The measured A^2^Π_1/2_−X^2^Σ^+^ (0, 0) band centre, ${\tilde{{\mathscr{T}}}}_{{\rm{e}}}$ = 13,287.8(1) cm^−1^ is in excellent agreement with the ab initio calculated value of 13,300(1,200) cm^−1^ (ref. ^32^). In accordance with theoretical predictions^6^, we found vibronic transitions with Δ*v* = 0 to be much stronger than those with Δ*v* = ±1. For most of the measurements, the power density used for the resonant step was 100(5) μJ cm^−2^ per pulse, as measured at the entry window of the beam line. Reducing the power by 50% did not reduce the resonant ionization rate, indicating that these transitions were measured well above saturation. The much weaker vibrational transitions with Δ*v* = ±1 were scanned with a pulsed dye laser of 500(5) μJ cm^−2^ power density per pulse (bandwidth of 0.1 cm^−1^). The Δ*v* = ±1 transitions were measured well above saturation and with laser beams of different characteristics, and so a precise estimation of the Franck–Condon factors could not be obtained. Instead, a lower limit of 0.97 for the peak intensity ratio *I*(0, 0)/*I*(0, 1) was derived, indicating highly diagonal Franck–Condon factors, an essential property for laser cooling^6^.

By measuring the resonant ionization rate for different time delays between the excitation and ionization laser pulses, we obtained an upper limit for the lifetime of the excited state ^2^Π_1/2_ (*v*′ = 0): *T*_1/2_ ≤ 50 ns. The measurements were performed with the wavenumber of the resonant laser fixed at the resonance value of the transition (*v*′, *v*″) = (0, 0). The resonant ionization rate dropped by more than 70% for delays above 50 ns. This short lifetime corresponds to a large spontaneous decay rate (>2 × 10^7^ s^−1^), which would allow for the application of strong optical forces for laser cooling. An additional concern for the suitability of laser cooling is related to the existence of metastable states lying energetically below the ^2^Π_1/2_ level, which could prevent the application of a closed optical-cooling loop, a major problem encountered for BaF (ref^6^.). In contrast to BaF, all other predicted electronic states (^2^Π_3/2_, ^2^Δ_3/2_ and ^2^Σ) in RaF were found to be energetically above the ^2^Π_1/2_ state, indicating that its electronic structure will allow for efficient optical-cooling cycles.

From combination differences of energetically low-lying vibronic transitions in the band system A^2^Π_1/2_−X^2^Σ^+^, we have derived experimental values for the harmonic frequency, ${\tilde{\omega }}_{{\rm{e}}}$, and the dissociation energies, ${\tilde{{\mathscr{D}}}}_{{\rm{e}}}$, using a Morse potential approximation. Results are given in Table 2, and further details of the analysis can be found in Methods section ‘Spectroscopic analysis’.

**Table 2:** ^226^RaF Morse potential parameters for X^2^Σ^+^ electronic ground and A^2^Π_1/2_ excited states

Parameter	${\tilde{{\boldsymbol{\omega }}}}_{{\bf{e}}}$ (cm^−1^)	${\tilde{\boldsymbol{\mathscr{D}}}}_{{\bf{e}}}$ (× 10^−4^ cm^−1^)
X^2^Σ^+^	441.8(1)	2.92(5)
A^2^Π_1/2_	435.5(1)	2.90(3)

**Fig. 3:**
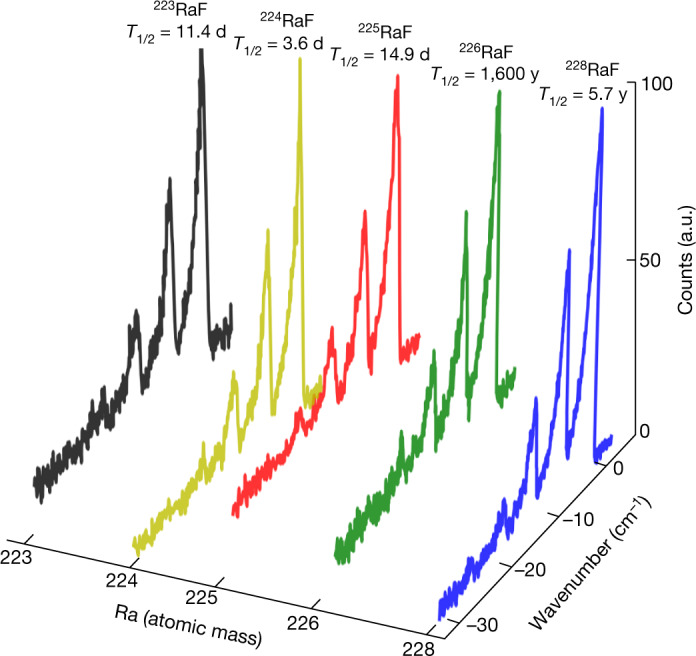
Vibronic spectra measured for different isotopologues of RaF. Measured vibronic absorption spectra for the A^2^Π_1/2_ ← X^2^Σ^+^ transition are shown for the isotopologues ^223^RaF, ^224^RaF, ^225^RaF, ^226^RaF and ^228^RaF. Wavenumber values are relative to the transition (0, 0) of ^226^RaF.

Furthermore, we measured the A^2^Π_1/2_ ← X^2^Σ^+^ vibronic spectra of ^226^RaF and the short-lived isotopologues ^223^RaF, ^224^RaF, ^225^RaF, and ^228^RaF (Fig. 3). All vibrational transitions were clearly observed, including those of the molecule with the shortest-lived radium isotope studied, ^224^RaF (*T*_1/2_ = 3.6 d). An on-line irradiation of the target material will enable the study of molecules containing isotopes with lifetimes as short as a few tens of milliseconds. The main limitation is dictated by the release from the target and the time spent in the RFQ trap (>5 ms). Future high-resolution measurements will enable studies of nuclear structure changes resulting from different isotopes and nuclear spins.

## Conclusions and future perspectives

In summary, this Article presents an experimental approach for performing laser spectroscopy studies of molecules containing radioactive nuclei, which are typically produced at rates lower than 10^6^ molecules s^−1^. Our results have established the energetically low-lying electronic structure of RaF, providing experimental evidence for the suitability of this diatomic molecule in a laser-cooling scheme. These findings are a pivotal step towards precision measurements in this system, which are expected to provide a highly sensitive environment for the exploration of physics beyond the Standard Model of particle physics.

Our experimental scheme can also be used to perform laser spectroscopy of a wide variety of neutral molecules and molecular ions, including those composed of isotopes with lifetimes of a few tens of milliseconds. Radioactive molecules can be precisely tailored to enhance their sensitivity to parity- and time-reversal-violating effects by introducing heavy and octupole-deformed nuclei. Moreover, by systematically replacing their constituent nuclei with different isotopes of the same element, both nuclear-spin-independent and nuclear-spin-dependent effects can be comprehensively studied. In addition, the present technique is applicable to other molecules of interest in studies of fundamental physics that are as yet experimentally unexplored, such as RaOH (ref. ^37^), RaO (ref. ^18^), RaH (ref. ^17^), AcF (ref. ^38^) and ^229^ThO (ref. ^5^).

In addition to the impact of our findings on quantum chemistry, nuclear structure and fundamental physics research, the ability to produce, mass-select and spectroscopically study short-lived radioactive molecules is of importance to other fields of research such as radiochemistry^21^ and astrophysics^39,40^. Laboratory measurements of the spectra of radioactive molecules of astrophysical interest will allow their unambiguous identification in future astronomical observations. Furthermore, the possibility of performing spectroscopy on fast molecular beams will enable sub-Doppler spectroscopy to be performed even on molecules created at high temperatures (>600 K). Thus, we expect our results will motivate further avenues of research at the increasingly capable radioactive-ion-beam facilities around the world.

## Methods

### Production of RaF molecules

Ra isotopes were produced 33 d before the laser-spectroscopy measurements by impinging 1.4 GeV protons on the cold UC_*x*_ target material. The target was exposed to pulses of 10^13^ protons per pulse over a period of 2 d. After irradiation with a total of 8 × 10^17^ protons, the target was kept in a sealed chamber filled with Ar gas. After day 33, the target was connected to the High-Resolution Separator (HRS) front-end at ISOLDE. FLUKA^41^ simulations predicted 2 × 10^13^ atoms of ^226^Ra in the target material (7.5 × 10^−9^ g), following proton irradiation of a cold target. The target was pumped down to pressures below 10^−5^ mbar, and the target holder and ion source were gradually heated up to about 1,300 °C, in order for the Ra isotopes to diffuse towards the surface of the target material. A leak valve attached to the target was used to inject CF_4_ into the target environment. The CF_4_ molecules dissociate and react with atoms and molecules on the target surface until an equilibrium is reached. RaF molecules were formed by reactive collisions of CF_4_ molecules with Ra atoms present inside the irradiated target material.

According to thermodynamic equilibrium calculations^42^, RaF_2_ or RaF are expected to form, depending on the local temperature. Within the temperature gradient between the target (1,300 °C) and the ion source (2,000 °C), RaF_2_ fully reacts to form RaF. A measured ratio of the ion-beam intensity of Ra^+^ to RaF^+^ of less than 0.05 indicates that more than 95% of the Ra isotopes released from the target material are converted and extracted as molecules.

**Extended Data Fig. 1:**
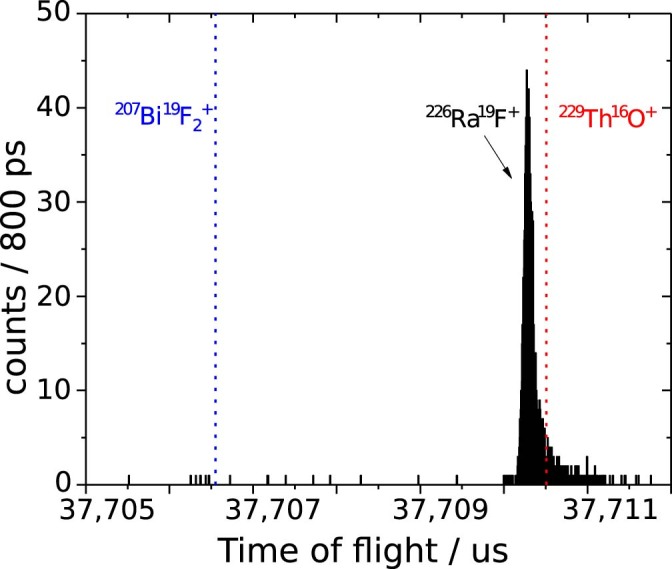
Time-of-flight spectrum measured at mass *A* = 245. The time-of-flight spectrum of the ^226^RaF^+^ (*A* = 245) beam as delivered from ISOLDE after 1,000 revolutions in the multi-reflection time-of-flight mass spectrometer. A mass resolving power of 1.7 × 10^5^ was achieved, which allowed the isobaric beam composition to be analysed. Only ^226^Ra^19^F^+^ ions were detected. The positions of the most probable accompanying ions are highlighted by dotted vertical lines. Source Data

The ^226^RaF^+^(*A* = 245) beam extracted from the ISOLDE target unit was sent to the ISOLTRAP setup^43^, where the molecular ions were captured, cooled and bunched by a different RFQ trap and subsequently analysed using a multi-reflection time-of-flight mass spectrometer^44^. A measured mass spectrum is shown in Extended Data Fig. 1. After 1,000 revolutions in the device, a mass resolving power (*R* = *m*/Δ*m*) of 1.7 × 10^5^ was achieved, which allowed the isobaric beam composition to be analysed. The only mass peak detected was identified as the signal of ^226^Ra^19^F^+^, confirming the purity of the beam from ISOLDE.

The intensity of RaF^+^ molecules depends strongly on the target and ion source temperature. For a target temperature of 1,300 °C, a mean value of 2 × 10^7^ molecules s^−1^ of ^226^RaF^+^ was measured after the mass separator. Depending on the molecular mass and beam intensity, the transmission efficiency through the RFQ trap varied from 15% to 30%. The ion-beam transmission from the ion trap to the interaction region was measured to be 25(5)%. The charge exchange cell vapour was heated to produce a measured neutralization rate of 30(5)%. Thus, we estimate that on average 5 × 10^4^ neutral ^226^RaF molecules s^−1^ were delivered to be resonantly excited. From the analysis of the measured spectra it was concluded that the neutral molecules populate the low-lying vibrational states *ν* = 0, 1, 2, 3, 4 following a relative population of 0.47:0.29:0.13:0.05:0.03. Resonantly ionized molecules with rates of the order of 10^3^ counts s^−1^ at the peak of the 0 ← 0 transition were measured at the particle detector. Future production of RaF^+^ molecular rates of the order of 10^9^–10^10^ molecules s^−1^ is feasible using active proton irradiation^45^.

### Laser setup

The resonance ionization schemes used for the study of RaF molecules are shown in Fig. 1. Three different laser systems were prepared to cover the scanning range from 12,800 cm^−1^ to 13,800 cm^−1^: 1) A dye-laser system (Dye1; Spectrolase 4000, Spectron) provided pulses of 100(5) μJ with a linewidth of 10 GHz (0.3 cm^−1^). 2) A dye laser (Dye2; Cobra, Sirah) with a narrower linewidth of 2.5 GHz (0.09 cm^−1^) produced pulses of similar energy. The lasers were loaded with either Styryl 8 or DCM dyes to provide wavenumber ranges 12,800–14,000 cm^−1^ and 15,150–16,600 cm^−1^, respectively. Both dye lasers were pumped by 532-nm pulses at 100 Hz, obtained from two different heads of a twin-head Nd:YAG laser (LPY 601 50–100 PIV, Litron). 3) A grating Ti:sapphire laser system with a linewidth of 2 GHz (0.07 cm^−1^) produced pulses of 20(1) μJ, pumped by 532-nm pulses at 1 kHz from a Nd:YAG laser (LDP-100MQ, LEE Laser). The non-resonant ionization step was obtained by 355-nm pulses of 30 mJ at 100 Hz, produced by the third-harmonic output of a high-power Nd:YAG laser (TRLi 250-100, Litron).

The release of the ion bunch was synchronized with the laser pulses by triggering the flash-lamps and Q-switch of the pulsed lasers with a digital delay pulse generator (Quantum Composers 9528).

The dye-laser wavelengths were measured with a wavelength meter (WS6-600 HighFinesse) and the Ti:sapphire laser wavelengths were measured by a wavelength meter (WSU-2 HighFinesse) calibrated by measuring a reference wavelength provided by a stabilized diode laser (DLC DL PRO 780, Toptica).

### Collinear and anti-collinear excitation

For the initial peak searching, a zero-degree mirror at the end of the beam line was used to reflect the laser light anti-collinearly with respect to the travelling direction of the RaF bunch. Thus, each scanning laser covered two different wavenumber regions in the molecular rest frame, owing to the Doppler shift present for the fast RaF molecules. For a molecule travelling at velocity *v*, the laser wavenumber in the laboratory frame, ${\tilde{\nu }}_{0}$, is related to the wavenumber in the molecule rest frame, $\tilde{\nu }$, by the expression $\tilde{\nu }=\tfrac{1+\beta \cos \,\theta }{\sqrt{1-{\beta }^{2}}}{\tilde{\nu }}_{0}$, with *β* = *v*/*c* (*c*, speed of light in vacuum) and where *θ* is the angle between the direction of the laser beam and the velocity of the molecule. For RaF molecules at 39,998(1) eV (*v* ≈ 0.18 m μs^−1^), a difference of 15.7 cm^−1^ is obtained between the laser pulse sent out collinearly (cos*θ* = 1) and anti-collinearly (cos*θ* = −1) with respect to the direction of the velocity of the molecule.

### Spectroscopic analysis

The peaks in the different spectra were identified by rebinning the spectra using coarse bin sizes with values up to 1 cm^−1^. Only groups of data points that were consistently observed with a 5-sigma significance above background were considered as candidates for transitions. The vibrational transitions in Fig. 2 show asymmetric line profiles with a maximum located towards higher wavenumbers. The band centres cannot be determined directly from the measured line profiles, and so we used the wavenumber positions of the maxima in our data analysis. Extended Data Table 1 lists the maximum peak positions and estimated uncertainties are given in parentheses. The wavenumber difference, $\Delta \tilde{\nu }$, of vibrational levels in the electronic ^2^Σ^+^ ground state and in the ^2^Π_1/2_ excited state were derived from combination differences of the recorded ^226^RaF spectra (see Extended Data Table 1).

In our analysis we used vibrational energy terms *E*_*v*_/(*hc*) of a Morse potential according to:

1 $${E}_{v}/(hc)={\tilde{\omega }}_{{\rm{e}}}\left(v+\frac{1}{2}\right)-\frac{{\tilde{\omega }}_{{\rm{e}}}^{2}}{4{\tilde{{\mathscr{D}}}}_{{\rm{e}}}}{\left(v+\frac{1}{2}\right)}^{2}$$

Energy-level differences

2 $$({E}_{v+1}-{E}_{v})/(hc)={\tilde{\omega }}_{{\rm{e}}}-\frac{{\tilde{\omega }}_{{\rm{e}}}^{2}}{2{\tilde{{\mathscr{D}}}}_{{\rm{e}}}}(v+1)$$

were used to derive the Morse potential parameters ${\tilde{\omega }}_{{\rm{e}}}$ and ${\tilde{{\mathscr{D}}}}_{{\rm{e}}}$ from a least-squares fit analysis. The derived energy-level differences are given in Extended Data Table 1, whereas Extended Data Table 2 contains the molecular parameters from the fit. The harmonic vibration frequencies ${\tilde{\omega }}_{{\rm{e}}}$ of the ^2^Σ^+^ and ^2^Π_1/2_ states are almost identical and correspond well to the theoretical predictions with a deviation of less than 5%; see Extended Data Table 2. The same holds for the estimated dissociation energy ${\tilde{{\mathscr{D}}}}_{{\rm{e}}}$, which is in better agreement with the values of ref. ^6^, as therein also the low-energy part of the potentials was used to estimate the dissociation energy.

In the case of the two low-lying ^2^Π fine-structure levels, the observed origins *T*_0,0_ agree well with the calculated values based on the Relativistic Correlation Consistent – Atomic Natural Orbital (RCC-ANO) basis set. From the energy difference of the fine-structure components the effective spin-orbital coupling parameter *A* is derived. For the ^2^Π states, the experimental value of 2,068(5) cm^−1^ is in good agreement with the calculated value. The band origins are in reasonable agreement with results from the RCC-ANO basis set calculation, if one attributes the Ω = 3/2 levels, which were computationally found to be of mixed Π_3/2_ and Δ_3/2_ character in this order of energies. A reverse assignment also gives better agreement with experiment. Calculations of the gas-phase bond lengths, dissociation energies and additional properties of RaF molecules have been reported^8,32,35^.

## Online content

Any methods, additional references, Nature Research reporting summaries, source data, extended data, supplementary information, acknowledgements, peer review information; details of author contributions and competing interests; and statements of data and code availability are available at 10.1038/s41586-020-2299-4.

### Source data

Source Data Extended Data Fig. 1

## Figures and Tables

**Extended Data Table 1:**
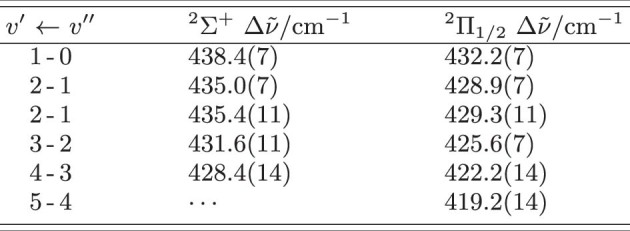
^226^RaF vibrational transitions in the electronic X^2^Σ^+^ ground state and the A^2^Π_1/2_ excited state derived from combination differences

Combined statistical and systematic uncertainties are given in parentheses.

**Extended Data Table 2:**
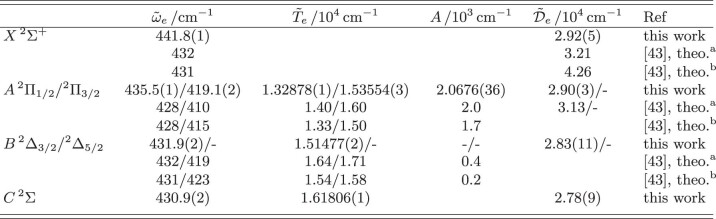
Molecular parameters of RaF from vibrational analysis of the electronic ground state (X^2^Σ^+^) and excited states (A^2^Π, B^2^Δ and C^2^Σ^+^)

^a^These calculations used Fock space coupled cluster singles and doubles, the Dyall basis set and a smaller active space.

^b^These calculations used Fock space coupled cluster singles and doubles, the RCC-ANO basis set and a larger active space.

Experimental results are compared with theoretical predictions^43^, theo.

For a direct comparison, theoretical values given for ${\tilde{\omega }}_{{\rm{e}}}$ should be scaled by $\sqrt{{m}_{{\rm{p}}}/u}$ ≈ 1.0036 to account for the atomic mass constant instead of the proton mass, *m*_p,_ in atomic mass units (*u*).

## Data Availability

Examples of vibronic spectra measured for RaF molecules are included as source data with this Article. All other relevant data supporting the findings of these studies are available from the corresponding author upon request.
